# Functional and structural characteristics of bacterial proteins that bind host cytokines

**DOI:** 10.1080/21505594.2017.1363140

**Published:** 2017-08-25

**Authors:** Martin Högbom, Riikka Ihalin

**Affiliations:** a Department of Biochemistry and Biophysics, Stockholm University, Stockholm, Sweden; b Department of Biochemistry, University of Turku, Turku, Finland

**Keywords:** bacterial cytokine-binding proteins, human pathogens, structural biology, virulence factor

## Abstract

Several human pathogens bind and respond to host cytokines, which can be considered a virulence mechanism that communicates defensive actions of the host to the pathogen. This review summarizes the current knowledge of bacterial cytokine-binding proteins, with a particular focus on their functional and structural characteristics. Many bacterial cytokine-binding proteins function in the development of infection and inflammation and mediate adhesion to host cells, suggesting multiple roles in pathogen-host interactions. The regions of the bacterial proteins that interact with host cytokines can display structural similarities to other proteins involved in cytokine signaling. However, there appears to be no central shared structural themes for bacterial cytokine-binding proteins, and they appear to possess structures that are different from the cytokine receptors of the host. Atomic-level information regarding receptor-cytokine interactions is needed to be able to disrupt these interactions and to elucidate the specific consequences of cytokine binding in a pathogen and host.

## Introduction

Certain bacteria, primarily opportunistic human pathogens, are able to bind host cytokines^1-7^ and respond to them by increasing their growth,^1,8,9^ forming biofilms^10,11^ or changing virulence characteristics.^5,6,12^ Although this type of interkingdom signaling has been recognized for over 2 decades,^1^ surprisingly few bacterial cytokine receptors have been identified to date.^2,4-7^ However, the known bacterial cytokine-binding proteins represent a rather versatile group of proteins, including a channel-forming usher protein,^2^ a gram-negative secretin,^6^ an outer membrane pore protein,^5^ a pilus subunit,^6^ an intrinsically disordered outer membrane lipoprotein,^13^ and a secreted protein displaying structural similarity to human cytokine receptors.^7^ In addition to bacteria, viruses have also been reported to possess receptors for host cytokines (for a review, see ref. ^14^). However, some of the viral receptors are thought to be co-opted from the host genome,^15^ thus representing a different protein family from the bacterial cytokine-binding proteins. The aim of this review is to provide an overview of the current knowledge of bacterial cytokine-binding proteins with a focus on their functional and structural characteristics. This analysis may help us understand the evolutionary development of this potential virulence feature of opportunistic pathogens and provide new insights into the development of novel anti-virulence agents that pose less selective pressure for antimicrobial resistance.

## Channel-forming outer membrane proteins

**Figure 1.**
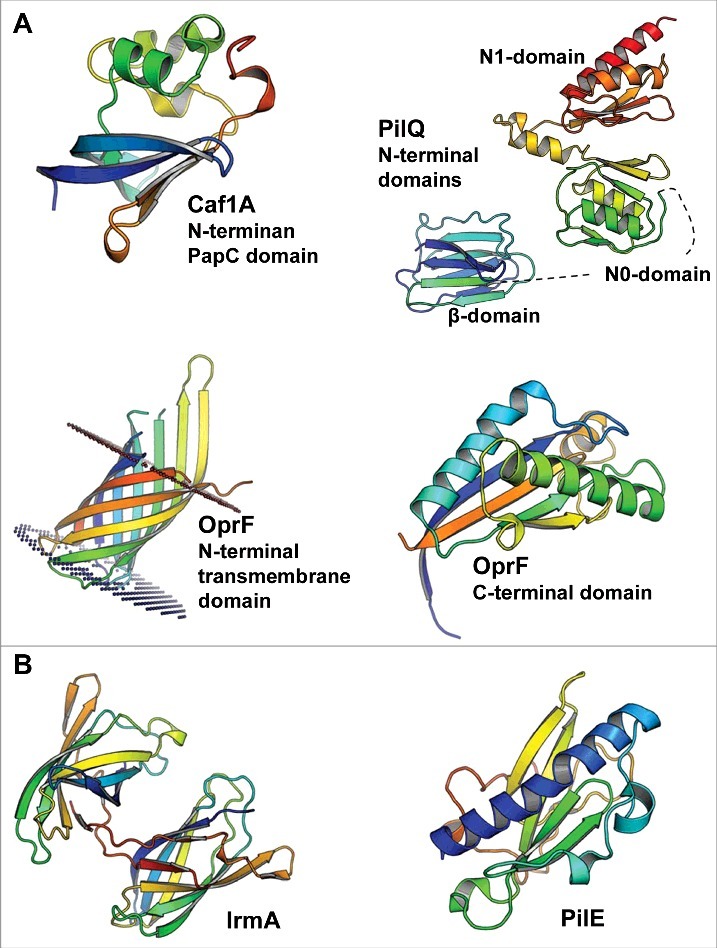
The 3D structures of bacterial cytokine-binding proteins colored from the N-terminus (blue) to the C-terminus (red). (A) The N-terminal PapC domain of *Y. pestis* Caf1A (PDB:4BOE), the β, N0 and N1 domains of *N. meningitidis* PilQ (PDB:4AV2), the N-terminal transmembrane domain (PDB:4RLC), and the C-terminal domain (PDB:5U1H) of *P. aeruginosa* OprF are located in the outer membrane of gram-negative species. (B) *E. coli* IrmA (PDB:5EK5) and *N. meningitidis* PilE (PDB:5JW8) are secreted to and face the extracellular space, respectively. The figures were prepared with PyMol (www.pymol.org).

Channel-forming outer membrane proteins (OMP) represent the oldest and the largest group of known bacterial cytokine-binding proteins (Fig. 1A). The first identified bacterial protein that interacted with high affinity with human interleukin-1β was the capsule antigen F1 assembly (Caf1A) protein of *Yersinia pestis*.^2^ Caf1A is an outer membrane usher protein that mediates the construction of the Caf1 fiber that forms the extracellular capsule.^16^ The capsule comprises solely of Caf1 proteins, which are exported to the outer membrane Caf1A usher protein, specifically to its N-terminal PapC domain,^17^ via the periplasmic Caf1M chaperone.^18,19^ Formation of the Caf1-Caf1M complex is critical for Caf1 fiber construction.^18^ The Caf1 subunit shares structural similarity with IL-β, suggesting potential interaction between IL-1β and Caf1A, which binds Caf1 during fiber secretion.^20^ Expression of Caf1A on the outer membrane of *E. coli* caused specific binding of ^125^I-labeled IL-1β to the cells; the Caf1-Caf1M complex inhibited this binding to Caf1A, indicating a common binding site.^2^ Thus, the IL-1β binding site in Caf1A overlaps with the binding site of the Caf1 capsular protein,^2^ which consists of the periplasmic N-terminal PapC domain of Caf1A^17^ (Fig. 1A). However, it has not been studied whether the binding of IL-1β to Caf1A prevents the binding of Caf1 to the PapC domain of Caf1A and subsequent capsule formation. As the binding of IL-1β to Caf1A-expressing *E. coli* cells was determined using a radiolabeled ligand,^2^ the method could not distinguish between extracellular and intracellular IL-1β. The N-terminal PapC domain of Caf1A is located in the periplasmic space, and it is thus possible that *Y. pestis* takes up the cytokine. As internalization of large intact host cytokines, such as the 17 kDa IL-1β (40 Å in diameter),^21^ may not be feasible, some researchers have hypothesized that the cytokines are first digested into smaller peptides before uptake.^8^ Gram-negative bacteria are able to take up host peptides, such as cationic α-helical antimicrobial peptides, and internalize them with the help of conserved lipoprotein Lpp.^22^ To obtain more specific information about the possible uptake of intact IL-1β, or its peptides, biotinylated cytokine or peptides can be used in combination with avidin-gold staining and transmission electron microscopy. The capsule antigen F1 is encoded by the 100-kb virulence plasmid pFra of *Y. pestis*, making it a unique feature of this species.^23^ The majority of natural *Y. pestis* strains possess the *caf* operon,^24^ indicating positive natural selection of F1 antigen. When considering the lifecycle of *Y. pestis* in its natural hosts (fleas and mammals), the *caf* operon is not essential for the transmission of the bacterium from fleas to mammals.^25^ The operon enhances virulence after transmission through a flea bite but is not needed for the development of bubonic plague.^25^ The F1 antigen, expressed at 37°C and secreted by Caf1A, forms a capsule around the pathogenic *Y. pestis*; the capsule inhibits adherence to macrophages, thus attenuating phagocytosis,^23^ and inhibits adhesion to human airway epithelial cells.^26^ Vaccination using F1 as the antigen protects mice against both bubonic and pneumonic plague,^27^ yet the virulence potential of F1 appears to differ between different mouse strains.^28^

Another bacterial outer membrane pore-forming protein that interacts with host cytokines, in this case IL-8 and TNF-α, is the secretin channel PilQ of *Neisseria meningitidis*.^6^ Although the exact ligand-binding site of PilQ is not known, the membrane protein displays functional similarity with Caf1A as it has a role in the formation and secretion of PilE pilus fibers.^29^ PilQ forms a pore consisting of 12 subunits.^30,31^ In one subunit, the interdomain space of the 2 N-terminal β-domains, which precede the N0 and N1 domains (Fig. 1A), contains 4 to 7 copies of an octapeptide small basic repeat (SBR) sequence PAKQQAAA, which affects pilus expression efficiency.^29^ As indicated by the name, the β-domains are rich in β-strands that stack together in sandwich structures.^31^ PilQ interacts with the PilE subunit both with its N-terminal and C-terminal regions and responds dynamically to these interactions.^32^ The PilQ secretin channel of *N. meningitidis*, which is involved in type IV pilus assembly, is differentially expressed depending on the surrounding host environment.^33^ PilQ expression is increased in cerebrospinal fluid (CSF) compared with the expression levels in blood and the nasal mucosa.^33^ Although less PilQ is expressed in the nasal mucosa than in the CSF, PilQ was shown to play a role in the adherence of *N. meningitidis* to a nasopharyngeal cell line.^33^ Moreover, *N. meningitidis* seems to suppress phagocytosis by macrophages in a PilQ-dependent manner, whereas PilQ increases the susceptibility of the bacterium to complement-mediated killing.^33^ *N. meningitidis* PilQ is also involved in the initial binding of the bacterium to the blood-brain barrier via interaction with the laminin receptor (LR). This interaction also involves another outer membrane pore-forming protein, PorA, as only Δ*pilQ*Δ*porA* double mutants demonstrate significantly reduced binding to the LR.^34^

The third OMP involved in the binding and response to cytokines is the nonspecific porin OprF of *Pseudomonas aeruginosa*.^5^ This porin binds interferon (IFN)-γ specifically, resulting in increased type I *P. aeruginosa* (PA-I) lectin expression, one of the major virulence factors of *P. aeruginosa*.^5^ OprF has a slightly different conformation and function than Caf1A and PilQ. Unlike Caf1A and PilQ, it does not form multimers in the outer membrane but instead may adopt 2 alternate conformations: a closed form with a periplasmic C-terminal α-helix-rich domain (Fig. 1A) and an open form with one β-barrel-forming domain.^35^ In addition to permitting the entrance of small ionic and polar molecules with a cutoff size smaller than 1.5 kDa,^36^ OprF plays a role in adhesion to human lung epithelial cells,^37^ biofilm development,^38^ and the sensing of quorum sensing (QS) signals.^39^ OprF is also involved in adhesion to and cytotoxicity against primary rat glial cells and immortalized human intestine cells, measured by LDH release.^39^ Moreover, the Δ*oprF* strain has a reduced ability to kill *Caenorhabditis elegans*, which can be used as an infection model for *P. aeruginosa*.^39^ The reduced cytotoxicity is due to the decreased expression levels of the type III secreted effector proteins ExoS and ExoT as well as virulence determinants, including exotoxin A, lectin PA-1L, pyocyanin and elastase.^39^ OprF is also a potential vaccine antigen as it induced both systemic and lung immunity in mice.^40^

## Proteins facing/in the extracellular space

In addition to channel-forming OMPs, 3 additional extracellular or extracellular space-facing bacterial proteins are involved in cytokine binding in some gram-negative species: one is an outer membrane lipoprotein, the second is secreted as a soluble protein, and the third forms type IV pili (Fig. 1B). These bacterial proteins, namely, the *Aggregatibacter actinomycetemcomitans* bacterial interleukin receptor I (BilRI), *Escherichia coli* interleukin receptor mimic protein A (IrmA), and the PilE subunit of *Neisseria meningitidis*, share some functional similarities.

*A. actinomycetemcomitans* is an oral opportunistic pathogen that is also able to cross the blood-brain barrier and cause abscesses in the brain.^41^ It possesses an outer membrane lipoprotein that interacts with various host cytokines *in vitro*, including IL-8, IL-1β, IL-10, TNF-α, TGF-1β, and IFN-γ.^13^ Unlike other bacterial cytokine-binding proteins identified thus far, the lipoprotein BilRI^4^ does not have a stable 3-dimensional fold without a binding ligand.^13^ In other words, BilRI is intrinsically disordered, which most likely accounts for its ability to bind multiple ligands. In addition to its outer membrane location,^4^ BilRI has been identified in *A. actinomycetemcomitans* outer membrane vesicles,^42^ which indicates that it may exert its function further away from the bacterial surface, as described below for IrmA.

The 3-dimensional structure of IrmA resembles the immunoglobulin (Ig)-like domain of fibronectin III and forms a stable dimer in solution after a domain swap (Fig. 1B).^7^ This dimer has structural similarity with the extracellular binding domains of human cytokine receptors IL-2R and IL-4R and to lesser extent with IL-10R. Indeed, IrmA has been shown to interact with the corresponding cytokines IL-2, IL-4 and IL-10.^7^ Typically, the structural conformation involved in the formation of the IrmA dimer is more common in the formation of fibers such as those in the Ig-fold of fimbrial subunits.^43^ The extracellular protein IrmA was first identified in studies seeking novel vaccine antigens to protect against *E. coli* sepsis in mice.^44^ Later, it was found that IrmA is regulated by the stress response protein OxyR, which represses the transcription of IrmA together with biofilm-associated antigen in uropathogenic *E. coli* (UPEC).^7^ The amino acid sequence of IrmA is conserved, with 97% identity between strains, and the gene encoding IrmA can be found in most sequenced UPEC strains.^7^ The fact that the serum of urosepsis patients infected with IrmA-positive UPEC strains has higher levels of IrmA-specific antibodies than the serum of healthy controls shows that IrmA has immunogenic potential *in vivo*.^7^

The type IV pili of *N. meningitidis* are composed of PilE subunits (Fig. 1B), which associate via interactions between different globular domains, between the long α-helixes, and between adjacent α-helixes and the globular domains.^45^ This differs slightly from the formation of the Caf1 fiber, in which the β-strand of one subunit complements the incomplete Ig-fold of the adjacent subunit,^46^ resembling the formation of IrmA dimer. However, the 3-dimensional structure of the PilE peptide backbone (Fig. 1B) may not be critical for the binding of host cytokines IL-8 and TNF-α to the pili as glycosylation of the pili was shown to play an important role in pilus interactions with cytokines that resemble lectins.^6^ Type IV pili are also involved in the crossing of the blood-brain barrier by *N. meningitidis*.^47,48^ During this central event in bacterial meningitis, type IV pili interact with 2 host proteins, namely, the endothelial receptors CD147 when adhering the host cells^47^ and β2 adrenergic receptor (β2AR) when inducing endothelial cell signaling needed for the disruption of the blood-brain barrier.^48^ The approximately 20 amino acid long surface-exposed hypervariable region of the PilE subunit plays a role in these interactions with the host endothelial cells.^49^ The glycosylated sites, which are most likely involved in the interaction with the host cytokines IL-8 and TNF-α, are located on the opposite site of the globular domain of PilE.^45^

## Role of bacterial cytokine-binding proteins in cytokine sensing

*N. meningitidis* PilQ is involved in internalization of IL-8 and TNF-α by the bacterium, which leads to altered expression of approximately 20% and 45% of the bacterial genome, respectively.^6^ These cytokines affect expression of proteins involved in cell membrane formation and function, bacterial survival and energy metabolism, indicating potential changes in bacterial virulence, such as resistance to complement killing.^6^ If PilQ functions as a channel for cytokine entry into the cell, the glycosylated PilE protein, which polymerizes to form the pili and mediates twitching motility, could guide the cytokines into the cell. Although the 3-dimensional structures of the PilQ channel^31^ and PilE pili^45^ have been solved, the molecular details regarding how the large cytokines IL-8 and TNF-α are accommodated with pili and are transported through the PilQ channel are lacking. The maximal diameters of an IL-8 dimer and a TNF-α trimer are approximately 40 Å^50^ and 50 Å,^51^ respectively. Thus, both of these cytokines can fit in the PilQ channel, which has a cavity of 55 Å in diameter.^31^ When internalized by *N. meningitidis*, TNF-α binds genomic DNA at several sites, including the promoter regions of PptB transferase (*pptB*), which modifies type 4 pili, adhesion and penetration protein (*app*) and meningococcal serine protease A (*mspa*), thereby increasing their expression.^6^ *N. meningitidis* mutants unable to express PilQ or glycosylated PilE result in reduced mortality in infected mice, though they can stimulate expression of host cytokines.^6^ Because the expression levels of *pptB, app* and *mspa* were lower in the mutant strains, the authors concluded that the enhanced animal survival observed was due to impaired response of the pathogen to host cytokines.^6^ However, glycosylation of PilE, the smallest difference between the wild type and mutant strains, might have roles in the virulence of *N. meningitidis* other than with regard to efficient binding of host cytokines. Thus, more specific information about atomic-level interactions between the cytokines and bacterial proteins is needed to identify ways to explicitly inhibit these interactions and to study the role of cytokine sensing in bacterial virulence.

When the opportunistic human pathogen *P. aeruginosa* binds IFN-γ with its OM OprF porin protein, PA-I lectin expression is induced to levels comparable to the activation potential of the QS signal C_4_ –homoserine lactone (C_4_-HSL).^5^ A link between the QS system and IFN-γ sensing was identified by the observation that exposure to IFN-γ led to *rhlI* gene expression and increased production of C_4_-HSL. When IFN-γ activated the QS system, it also enhanced the production of virulence factor pyocyanin,^5^ a secondary metabolite, which most likely is required for the full virulence of *P. aeruginosa* in lung infections.^52^ Although OprF forms a pore in the outer membrane, it seemingly does not function as a channel for IFN-γ entry into the bacterial cells, as only the membrane fraction of *P. aeruginosa* and not the soluble cytosolic fraction interacted with IFN-γ.^5^ Moreover, IFN-γ was the only cytokine that bound to the *P. aeruginosa* membrane and induced the production of PA-I lectin,^5^ a central virulence determinant, which increases the toxicity of *P. aeruginosa* exotoxins in experimental gut-derived sepsis, impairing the function of epithelial cells.^53^

Because the secreted cytokine-binding protein IrmA of *E. coli* interacts and sequesters its ligands IL-2, IL-4, and possibly also IL-10 in the extracellular environment,^7^ it is likely that this bacterial cytokine receptor does not mediate any signaling in the bacterial cells. However, IrmA may interfere with the proper cytokine immune defense function in the host. The main functions of the major cytokine ligands of IrmA (IL-2 and IL-4) are related to the production of antibodies, maturation of regulatory T-cells and proliferation and differentiation of natural killer cells (for reviews, see refs. ^54,55^). However, the effects of IrmA on host immune defense have not been studied in an infection model.

The first characterized bacterial cytokine receptor, Caf1A, was identified using recombinant protein techniques with heterologous expression in *E. coli*.^2^ Thus, the cytokine-sensing functions of Caf1A in its natural environment in the outer membrane of *Y. pestis* as well as the effects of IL-1β on the physiology of the host bacteria remain to be studied. However, as stated earlier in this review, the periplasmic location of the IL-1β interaction site of Caf1A in the outer membrane of *Y. pestis* suggests that Caf1A participates in the internalization of the cytokine, similar to PilQ in *N. meningitidis*.^6^

## Structural comparisons of bacterial cytokine receptors

**Figure 2.**
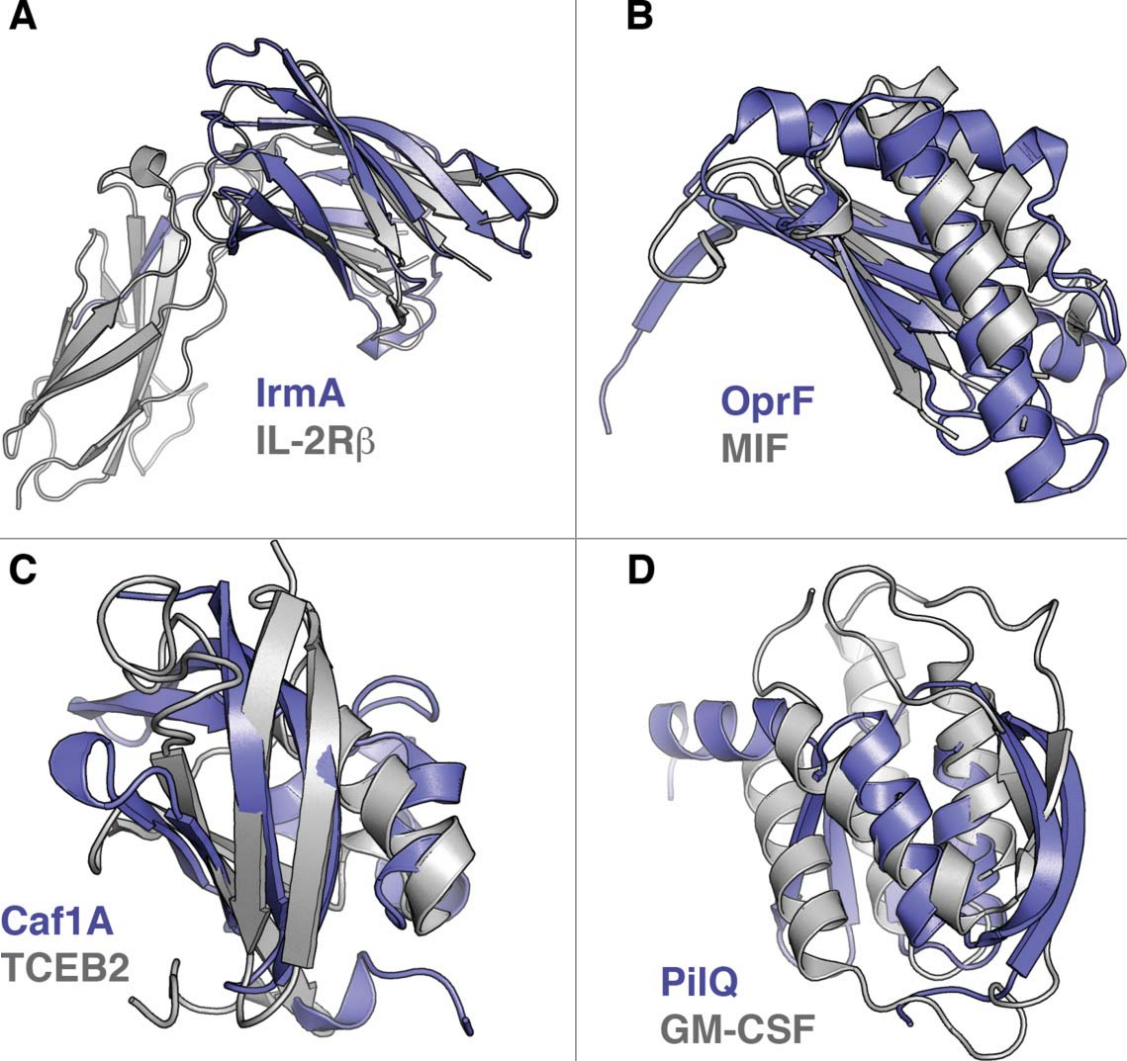
Structural similarities of bacterial cytokine-binding proteins and proteins involved in cytokine signaling. The bacterial proteins are shown in blue. (A) IrmA compared with the N-terminal Ig-fold domain of β-domain of IL-2R (PDB:2B5I), (B) the C-terminal domain of OprF compared with macrophage migration inhibitory factor (MIF) (PDB:4P7M), (C) the N-terminal PapC domain of Caf1A compared with transcription elongation factor b polypeptide 2 (TCEB2) (PDB:2IZV), and (D) the N0 domain of PilQ compared with human granulocyte macrophage colony stimulating factor receptor (GM-CSF) (PDB:5D71) are shown. Superimpositions were performed using the secondary-structure matching (SSM) tool in Coot.^59^ The figures were prepared with PyMol (www.pymol.org).

The bacterial cytokine-binding proteins for which structures are available are structurally diverse and show no general structural similarity (Fig. 1). This is not surprising, as a majority binds structurally different cytokines. It should be noted that when specifically comparing the PilE and PilQ proteins of *N. meningitidis*, which are reported to bind to similar cytokines, no structural similarity was detected. This comparison is naturally limited to the domains of the PilQ protein that have been structurally characterized. However, no significant sequence similarity is observed between PilE and any part of the PilQ protein, consistent with the lack of structural similarity. One central question regarding inter-kingdom (host-pathogen) signaling or protein-protein interactions is whether the proteins involved are evolutionarily related and, if so, in what way. As structure is generally more conserved than sequence, we sought to investigate whether bacterial cytokine-binding proteins show structural similarities to canonical cytokine receptors or proteins otherwise involved in cytokine signaling. To investigate this question, we searched for structural similarities between the bacterial cytokine-binding proteins and proteins involved in signaling for which high-resolution structures are available. Searches were performed using the protein structure comparison service PDBeFold at the European Bioinformatics Institute (http://www.ebi.ac.uk/msd-srv/ssm)^56^ against a set of approximately 2000 PDB entries of cytokine-related structures. Interestingly, no strong or highly significant structural similarities were identified. This observation suggests a relationship that is not direct, at least with regard to relatively recent horizontal gene transfer. However, 4 of the bacterial proteins displayed weak structural similarities to cytokine-related proteins (Fig. 2). IrmA shows structural similarity to the N-terminal Ig-fold domain of the β-domain of IL-2R (PDB:2B5I) despite a low sequence similarity (Fig. 2A), and the C-terminal periplasmic domain of OprF^35^ shows some structural similarity to macrophage migration inhibitory factor (MIF) (PDB:4P7M), which is a broadly expressed pro-inflammatory cytokine (note that in this example the bacterial protein is actually similar to a cytokine and not a cytokine receptor) (Fig. 2B). Moreover, the N-terminal PapC domain of Caf1A shows some similarity to transcription elongation factor b polypeptide 2 (TCEB2) (PDB:2IZV) (Fig. 2C), and the N0 domain of PilQ is partially similar to human granulocyte macrophage colony-stimulating factor receptor (GM-CSF) (PDB:5D71), a known cytokine receptor (CD116; Fig. 2D). As mentioned above, the identified structural similarities are very weak, and it remains to be determined whether these similarities are also reflected in target binding geometries. However, only IL-2R interacts with the same binding ligand, *i.e.*, IL-2, as the structurally similar bacterial protein IrmA. MIF, which shares some structural characteristics with IFN-γ binding OprF, interacts with human CXCR2, CXCR4 and DC74 receptors (for a review, see ref. ^57^), without any known linkage to IFN-γ. Moreover, the intracellular TCEB2 or GM-CSF receptor is not known to interact with IL-1β, similar to the N-terminal PapC domain of Caf1A, or with IL-8 and TNF-1α, as with the N0 domain of PilQ, respectively.

## Conclusions

The cytokine-sequestering capacity of individual bacterial cells has not been studied in detail. The *E. coli* cells producing functional recombinant proteins of a major fraction of the *Y. pestis f1* operon, *i.e.*, Caf1M, Caf1 and Caf1A, shows approximately 10^4^ IL-1β binding sites per cell,^2^ yet information about the endogenous cytokine-binding capacity of different pathogens as well as the bacterial cytokine-sequestering efficiency is still missing. Atomic-level information about cytokine-bacterial protein interactions is needed to specifically disturb cytokine binding, which can be achieved, for instance, by producing bacterial strains harboring protein variants devoid of cytokine-binding capacity. Such modified strains can be used in infection models to shed light on the question of whether cytokine binding also affects host defense. Although we described in this review only bacterial proteins that interact directly with host cytokines, various pathogens, such as *Neisseria, Yersinia* and *Staphylococcus* species possess indirect methods of binding host chemokines. These pathogens have the ability to bind heparin and other sulfated polysaccharides, which in turn can sequester various host heparin-binding molecules, including IFN-γ, monocyte chemotactic protein 3 and macrophage inflammatory protein 1α.^58^ In these cases sequestering of chemokines has been shown to decrease chemokine-induced chemotaxis and increase the invasion potential of the pathogens.^58^

It is likely that the cytokine-binding capacity of individual cells varies widely in a bacterial cell population, as indicated by earlier studies,^5,6^ and this could be the normal situation, especially in bacterial biofilms. In addition to limited data regarding the cytokine-sequestering capacity of individual bacterial cells, surprisingly little is known about the cytokine-binding affinities of bacterial proteins. However, this could be easily studied if soluble recombinant bacterial proteins are available. Currently, various research methods can be exploited, starting from traditional microplate assays combined with cytokine-specific antibodies to more sophisticated methods, such as surface plasmon resonance, isothermal titration calorimetry and microscale thermophoresis.

Earlier studies aimed at elucidating the role of cytokine-binding proteins in the virulence of the bacterial pathogens have revealed functions related to the development of infection and inflammation (Table 1). Many bacterial cytokine-binding proteins are able to adhere to various host cells, including the binding necessary for crossing the blood-brain barrier, suggesting there are multiple roles for these bacterial proteins in pathogen-host interactions. Due to the relatively young age of the host cytokine-sensing field as mode of an interkingdom signaling as well as the narrow scope of the studied bacterial species, the clinical significance of host cytokine-binding as a biologic phenomenon requires more detailed studies in infection models. In a few cases, the regions of the bacterial proteins that interact with host cytokines display structural similarities with other proteins involved in canonical cytokine signaling. Whether this similarity is due to divergent or convergent evolution or due to the use of commonly used folds, such as the immunoglobulin fold, is currently unknown. However, there appear to be no central common structural themes for bacterial cytokine-binding proteins (Fig. 1), and they appear to include structures that are different from native cytokine receptors. This provides an interesting starting point for studying the evolution and function of inter-kingdom cytokine signaling. In particular, structures of complexes between bacterial cytokine-binding proteins and their interacting cytokines would be particularly informative. Atomic-level information about such interactions is needed to be able to disrupt these interactions and elucidate the specific consequences of cytokine binding to a pathogen and host. This is exceptionally important, as cytokine-binding bacterial proteins appear to have versatile roles in the virulence of pathogens.

**Table 1.** Cytokine-binding bacterial proteins, their ligands and their virulence properties.

Protein	Type	Species	Cytokine ligands	Effect of cytokine binding	Virulence	PDB^a^	Ref
Caf1A	OM usher	*Yp*^b^	IL-1β	Potential IL-1β uptake	F1 antigen secreted by Caf1A inhibits adhesion to macrophages and airway epithelia, potential vaccine antigen	4BOE (N-terminal PapC domain)	^2,17,23,5-27^
PilQ	OM secretin	*Nm*^c^	IL-8, TNF-α	Cytokine uptake, increased virulence, complement resistance	Adherence to nasopharyngeal cells, inhibits phagocytosis by macrophages, initial binding to the blood-brain barrier	4AV2 (Domains N0 and N1)	^6,31,33,34^
OprF	OM porin	*Pa*^d^	IFN-γ	Increased production of PA-I lectin and pyocyanin, activation of QS signaling	Adherence to gliad and intestine cells, cytotoxic, induction of other virulence factors, potential vaccine antigen	4RLC (N-terminal transmembrane domain) 5U1H (C-terminal domain)	^5,35,37-40^
IrmA	secreted	*Ec*^e^	IL-2, IL-4, IL-10	Not determined	Immunogenic, potential vaccine candidate	5EK5	^7,44^
PilE	Pilus subunit	*Nm*^c^	IL-8, TNF-α	Cytokine uptake, increased virulence, complement resistance	Adheres to endothelial cells in the blood-brain barrier, induces host cell signaling	5JW8	^6,45^^,^^45,47-49^
BilRI	OM lipo-protein	*Aa*^f^	IL-1β, IL-8, IL-10, TNF-α, TGF-1β, IFN-γ	IL-1β uptake, decreased amount of extracellular DNA in biofilm	Not determined	N/A	^4,13,42^

*Notes*.

a The Protein Data Bank

b Yersinia pestis

c Neisseria meningitidis

d Pseudomonas aeruginosa

e Escherichia coli

f Aggregatibacter actinomycetemcomitans

## References

[1] Porat R, Clark BD, Wolff SM, Dinarello CA Enhancement of growth of virulent strains of Escherichia coli by interleukin-1. Science. 1991;254:430-2. doi:10.1126/science.1833820. PMID:1833820 1833820

[2] Zav'yalov VP, Chernovskaya TV, Navolotskaya EV, Karlyshev AV, MacIntyre S, Vasiliev AM, Abramov VM Specific high affinity binding of human interleukin 1 beta by Caf1A usher protein of Yersinia pestis. FEBS Lett. 1995;371:65-8. doi:10.1016/0014-5793(95)00878-D. PMID:7664886 7664886

[3] Paino A, Lohermaa E, Sormunen R, Tuominen H, Korhonen J, Pöllänen MT, Ihalin R Interleukin-1beta is internalised by viable Aggregatibacter actinomycetemcomitans biofilm and locates to the outer edges of nucleoids. Cytokine. 2012;60:565-74. doi:10.1016/j.cyto.2012.07.024. PMID:22898394 22898394

[4] Paino A, Ahlstrand T, Nuutila J, Navickaite I, Lahti M, Tuominen H, Välimaa H, Lamminmäki U, Pöllänen MT, Ihalin R Identification of a novel bacterial outer membrane interleukin-1Beta-binding protein from Aggregatibacter actinomycetemcomitans. PLoS One. 2013;8:e70509. doi:10.1371/journal.pone.0070509. PMID:23936223 23936223 PMC3729834

[5] Wu L, Estrada O, Zaborina O, Bains M, Shen L, Kohler JE, Patel N, Musch MW, Chang EB, Fu YX, et al. Recognition of host immune activation by Pseudomonas aeruginosa. Science. 2005;309:774-7. doi:10.1126/science.1112422. PMID:16051797 16051797

[6] Mahdavi J, Royer PJ, Sjölinder HS, Azimi S, Self T, Stoof J, Wheldon LM, Brännström K, Wilson R, Moreton J, et al. Pro-inflammatory cytokines can act as intracellular modulators of commensal bacterial virulence. Open Biol. 2013;3:130048. doi:10.1098/rsob.130048. PMID:24107297 24107297 PMC3814720

[7] Moriel DG, Heras B, Paxman JJ, Lo AW, Tan L, Sullivan MJ, Dando SJ, Beatson SA, Ulett GC, Schembri MA Molecular and structural characterization of a novel Escherichia coli interleukin receptor mimic protein. MBio. 2016;7:e02046-15. doi:10.1128/mBio.02046-15. PMID:26980835 26980835 PMC4807369

[8] Kanangat S, Bronze MS, Meduri GU, Postlethwaite A, Stentz F, Tolley E, Schaberg D Enhanced extracellular growth of Staphylococcus aureus in the presence of selected linear peptide fragments of human interleukin (IL)-1beta and IL-1 receptor antagonist. J Infect Dis. 2001;183:65-9. doi:10.1086/317645. PMID:11076706 11076706

[9] Meduri GU, Kanangat S, Stefan J, Tolley E, Schaberg D Cytokines IL-1beta, IL-6, and TNF-alpha enhance in vitro growth of bacteria. Am J Respir Crit Care Med. 1999;160:961-7. doi:10.1164/ajrccm.160.3.9807080. PMID:10471625 10471625

[10] McLaughlin RA, Hoogewerf AJ Interleukin-1beta-induced growth enhancement of Staphylococcus aureus occurs in biofilm but not planktonic cultures. Microb Pathog. 2006;41:67-79. doi:10.1016/j.micpath.2006.04.005. PMID:16769197 16769197

[11] Paino A, Tuominen H, Jääskeläinen M, Alanko J, Nuutila J, Asikainen SE, Pelliniemi LJ, Pöllänen MT, Chen C, Ihalin R Trimeric form of intracellular ATP synthase subunit beta of Aggregatibacter actinomycetemcomitans binds human interleukin-1beta. PLoS One. 2011;6:e18929. doi:10.1371/journal.pone.0018929. PMID:21533109 21533109 PMC3078924

[12] Kanangat S, Postlethwaite A, Cholera S, Williams L, Schaberg D Modulation of virulence gene expression in Staphylococcus aureus by interleukin-1beta: Novel implications in bacterial pathogenesis. Microbes Infect. 2007;9:408-15. doi:10.1016/j.micinf.2006.12.018. PMID:17307379 17307379

[13] Ahlstrand T, Tuominen H, Beklen A, Torittu A, Oscarsson J, Sormunen R, Pöllänen MT, Permi P, Ihalin R A novel intrinsically disordered outer membrane lipoprotein of Aggregatibacter actinomycetemcomitans binds various cytokines and plays a role in biofilm response to interleukin-1beta and interleukin-8. Virulence. 2017;8:115-34. doi:10.1080/21505594.2016.1216294. PMID:27459270 27459270 PMC5383217

[14] Alcami A Viral mimicry of cytokines, chemokines and their receptors. Nat Rev Immunol. 2003;3:36-50. doi:10.1038/nri980. PMID:12511874 12511874

[15] Spriggs MK Cytokine and cytokine receptor genes ‘captured’ by viruses. Curr Opin Immunol. 1994;6:526-9. doi:10.1016/0952-7915(94)90136-8. PMID:7946038 7946038

[16] Karlyshev AV, Galyov EE, Smirnov OY, Guzayev AP, Abramov VM, Zav'yalov VP A new gene of the f1 operon of Y. pestis involved in the capsule biogenesis. FEBS Lett. 1992;297:77-80. doi:10.1016/0014-5793(92)80331-A. PMID:1551441 1551441

[17] Di Yu X, Dubnovitsky A, Pudney AF, Macintyre S, Knight SD, Zavialov AV Allosteric mechanism controls traffic in the chaperone/usher pathway. Structure. 2012;20:1861-71. doi:10.1016/j.str.2012.08.016. PMID:22981947 22981947

[18] MacIntyre S, Zyrianova IM, Chernovskaya TV, Leonard M, Rudenko EG, Zav'yalov VP, Chapman DA An extended hydrophobic interactive surface of Yersinia pestis Caf1M chaperone is essential for subunit binding and F1 capsule assembly. Mol Microbiol. 2001;39:12-25. doi:10.1046/j.1365-2958.2001.02199.x. PMID:11123684 11123684

[19] Zavialov AV, Kersley J, Korpela T, Zav'yalov VP, MacIntyre S, Knight SD Donor strand complementation mechanism in the biogenesis of non-pilus systems. Mol Microbiol 2002;45:983-95. doi:10.1046/j.1365-2958.2002.03066.x. PMID:12180918 12180918

[20] Zav'yalov V, Denesyuk A, Zav'yalova G, Korpela T Molecular modeling of the steric structure of the envelope F1 antigen of Yersinia pestis. Immunol Lett. 1995;45:19-22. doi:10.1016/0165-2478(94)00194-V. PMID:7542626 7542626

[21] Finzel BC, Clancy LL, Holland DR, Muchmore SW, Watenpaugh KD, Einspahr HM Crystal structure of recombinant human interleukin-1 beta at 2.0 Å resolution. J Mol Biol 1989;209:779-91. doi:10.1016/0022-2836(89)90606-2. PMID:2585509 2585509

[22] Chang TW, Lin YM, Wang CF, Liao YD Outer membrane lipoprotein Lpp is gram-negative bacterial cell surface receptor for cationic antimicrobial peptides. J Biol Chem 2012;287:418-28. doi:10.1074/jbc.M111.290361. PMID:22084237 22084237 PMC3249093

[23] Du Y, Rosqvist R, Forsberg A Role of fraction 1 antigen of Yersinia pestis in inhibition of phagocytosis. Infect Immun. 2002;70:1453-60. doi:10.1128/IAI.70.3.1453-1460.2002. PMID:11854232 11854232 PMC127752

[24] Meka-Mechenko TV F1-negative natural Y. pestis strains. Adv Exp Med Biol. 2003;529:379-81. doi:10.1007/0-306-48416-1_76. PMID:12756794 12756794

[25] Sebbane F, Jarrett C, Gardner D, Long D, Hinnebusch BJ The Yersinia pestis caf1M1A1 fimbrial capsule operon promotes transmission by flea bite in a mouse model of bubonic plague. Infect Immun. 2009;77:1222-9. doi:10.1128/IAI.00950-08. PMID:19103769 19103769 PMC2643634

[26] Liu F, Chen H, Galvan EM, Lasaro MA, Schifferli DM Effects of psa and F1 on the adhesive and invasive interactions of Yersinia pestis with human respiratory tract epithelial cells. Infect Immun. 2006;74:5636-44. doi:10.1128/IAI.00612-06. PMID:16988239 16988239 PMC1594889

[27] Titball RW, Williamson ED Vaccination against bubonic and pneumonic plague. Vaccine. 2001;19:4175-84. doi:10.1016/S0264-410X(01)00163-3. PMID:11457543 11457543

[28] Weening EH, Cathelyn JS, Kaufman G, Lawrenz MB, Price P, Goldman WE, Miller VL The dependence of the Yersinia pestis capsule on pathogenesis is influenced by the mouse background. Infect Immun. 2011;79:644-52. doi:10.1128/IAI.00981-10. PMID:21115720 21115720 PMC3028848

[29] Tonjum T, Caugant DA, Dunham SA, Koomey M Structure and function of repetitive sequence elements associated with a highly polymorphic domain of the Neisseria meningitidis PilQ protein. Mol Microbiol. 1998;29:111-24. doi:10.1046/j.1365-2958.1998.00910.x. PMID:9701807 9701807

[30] Collins RF, Frye SA, Kitmitto A, Ford RC, Tonjum T, Derrick JP Structure of the Neisseria meningitidis outer membrane PilQ secretin complex at 12 Å resolution. J Biol Chem. 2004;279:39750-6. doi:10.1074/jbc.M405971200. PMID:15254043 15254043

[31] Berry JL, Phelan MM, Collins RF, Adomavicius T, Tonjum T, Frye SA, Bird L, Owens R, Ford RC, Lian LY, et al. Structure and assembly of a trans-periplasmic channel for type IV pili in Neisseria meningitidis. PLoS Pathog. 2012;8:e1002923. doi:10.1371/journal.ppat.1002923. PMID:23028322 23028322 PMC3441751

[32] Collins RF, Frye SA, Balasingham S, Ford RC, Tonjum T, Derrick JP Interaction with type IV pili induces structural changes in the bacterial outer membrane secretin PilQ. J Biol Chem. 2005;280:18923-30. doi:10.1074/jbc.M411603200. PMID:15753075 15753075

[33] Liu Y, Zhang D, Engström A, Merenyi G, Hagner M, Yang H, Kuwae A, Wan Y, Sjölinder M, Sjölinder H Dynamic niche-specific adaptations in Neisseria meningitidis during infection. Microbes Infect. 2016;18:109-17. doi:10.1016/j.micinf.2015.09.025. PMID:26482500 26482500

[34] Orihuela CJ, Mahdavi J, Thornton J, Mann B, Wooldridge KG, Abouseada N, Oldfield NJ, Self T, Ala'Aldeen DA, Tuomanen EI Laminin receptor initiates bacterial contact with the blood brain barrier in experimental meningitis models. J Clin Invest. 2009;119:1638-46. doi:10.1172/JCI36759. PMID:19436113 19436113 PMC2689107

[35] Sugawara E, Nestorovich EM, Bezrukov SM, Nikaido H Pseudomonas aeruginosa porin OprF exists in two different conformations. J Biol Chem. 2006;281:16220-9. doi:10.1074/jbc.M600680200. PMID:16595653 16595653 PMC2846725

[36] Nestorovich EM, Sugawara E, Nikaido H, Bezrukov SM Pseudomonas aeruginosa porin OprF: Properties of the channel. J Biol Chem. 2006;281:16230-7. doi:10.1074/jbc.M600650200. PMID:16617058 16617058 PMC2846715

[37] Azghani AO, Idell S, Bains M, Hancock RE Pseudomonas aeruginosa outer membrane protein F is an adhesin in bacterial binding to lung epithelial cells in culture. Microb Pathog. 2002;33:109-14. doi:10.1006/mpat.2002.0514. PMID:12220987 12220987

[38] Yoon SS, Hennigan RF, Hilliard GM, Ochsner UA, Parvatiyar K, Kamani MC, Allen HL, DeKievit TR, Gardner PR, Schwab U, et al. Pseudomonas aeruginosa anaerobic respiration in biofilms: Relationships to cystic fibrosis pathogenesis. Dev Cell. 2002;3:593-603. doi:10.1016/S1534-5807(02)00295-2. PMID:12408810 12408810

[39] Fito-Boncompte L, Chapalain A, Bouffartigues E, Chaker H, Lesouhaitier O, Gicquel G, Bazire A, Madi A, Connil N, Veron W, et al. Full virulence of Pseudomonas aeruginosa requires OprF. Infect Immun. 2011;79:1176-86. doi:10.1128/IAI.00850-10. PMID:21189321 21189321 PMC3067511

[40] Krause A, Whu WZ, Xu Y, Joh J, Crystal RG, Worgall S Protective anti-Pseudomonas aeruginosa humoral and cellular mucosal immunity by AdC7-mediated expression of the P. aeruginosa protein OprF. Vaccine. 2011;29:2131-9. doi:10.1016/j.vaccine.2010.12.087. PMID:21215829 21215829 PMC3061442

[41] Rahamat-Langendoen JC, van Vonderen MG, Engström LJ, Manson WL, van Winkelhoff AJ, Mooi-Kokenberg EA Brain abscess associated with Aggregatibacter actinomycetemcomitans: Case report and review of literature. J Clin Periodontol. 2011;38:702-6. doi:10.1111/j.1600-051X.2011.01737.x. PMID:21539594 21539594

[42] Kieselbach T, Zijnge V, Granström E, Oscarsson J Proteomics of Aggregatibacter actinomycetemcomitans outer membrane vesicles. PLoS One. 2015;10:e0138591. doi:10.1371/journal.pone.0138591. PMID:26381655 26381655 PMC4575117

[43] Rose RJ, Welsh TS, Waksman G, Ashcroft AE, Radford SE, Paci E Donor-strand exchange in chaperone-assisted pilus assembly revealed in atomic detail by molecular dynamics. J Mol Biol. 2008;375:908-19. doi:10.1016/j.jmb.2007.10.077. PMID:18054959 18054959

[44] Moriel DG, Bertoldi I, Spagnuolo A, Marchi S, Rosini R, Nesta B, Pastorello I, Corea VA, Torricelli G, Cartocci E, et al. Identification of protective and broadly conserved vaccine antigens from the genome of extraintestinal pathogenic Escherichia coli. Proc Natl Acad Sci U S A. 2010;107:9072-7. doi:10.1073/pnas.0915077107. PMID:20439758 20439758 PMC2889118

[45] Kolappan S, Coureuil M, Yu X, Nassif X, Egelman EH, Craig L Structure of the Neisseria meningitidis type IV pilus. Nat Commun. 2016;7:13015. doi:10.1038/ncomms13015. PMID:27698424 27698424 PMC5059446

[46] Zavialov AV, Tischenko VM, Fooks LJ, Brandsdal BO, Aqvist J, Zav'yalov VP, Macintyre S, Knight SD Resolving the energy paradox of chaperone/usher-mediated fibre assembly. Biochem J. 2005;389:685-94. doi:10.1042/BJ20050426. PMID:15799718 15799718 PMC1180718

[47] Bernard SC, Simpson N, Join-Lambert O, Federici C, Laran-Chich MP, Maissa N, Bouzinba-Segard H, Morand PC, Chretien F, Taouji S, et al. Pathogenic Neisseria meningitidis utilizes CD147 for vascular colonization. Nat Med. 2014;20:725-31. doi:10.1038/nm.3563. PMID:24880614 24880614 PMC7095922

[48] Coureuil M, Lecuyer H, Scott MG, Boularan C, Enslen H, Soyer M, Mikaty G, Bourdoulous S, Nassif X, Marullo S Meningococcus hijacks a beta2-adrenoceptor/beta-arrestin pathway to cross brain microvasculature endothelium. Cell. 2010;143:1149-60. doi:10.1016/j.cell.2010.11.035. PMID:21183077 21183077

[49] Miller F, Phan G, Brissac T, Bouchiat C, Lioux G, Nassif X, Coureuil M The hypervariable region of meningococcal major pilin PilE controls the host cell response via antigenic variation. MBio. 2014;5:e01024-13. doi:10.1128/mBio.01024-13. PMID:24520062 24520062 PMC3950515

[50] Baldwin ET, Weber IT, St Charles R, Xuan JC, Appella E, Yamada M, Matsushima K, Edwards BF, Clore GM, Gronenborn AM Crystal structure of interleukin 8: Symbiosis of NMR and crystallography. Proc Natl Acad Sci U S A. 1991;88:502-6. doi:10.1073/pnas.88.2.502. PMID:1988949 1988949 PMC50839

[51] Eck MJ, Sprang SR The structure of tumor necrosis factor-alpha at 2.6 Å resolution. Implications for receptor binding. J Biol Chem. 1989;264:17595-605. PMID:2551905 2551905 10.2210/pdb1tnf/pdb

[52] Lau GW, Ran H, Kong F, Hassett DJ, Mavrodi D Pseudomonas aeruginosa pyocyanin is critical for lung infection in mice. Infect Immun. 2004;72:4275-8. doi:10.1128/IAI.72.7.4275-4278.2004. PMID:15213173 15213173 PMC427412

[53] Laughlin RS, Musch MW, Hollbrook CJ, Rocha FM, Chang EB, Alverdy JC The key role of Pseudomonas aeruginosa PA-I lectin on experimental gut-derived sepsis. Ann Surg. 2000;232:133-42. doi:10.1097/00000658-200007000-00019. PMID:10862206 10862206 PMC1421122

[54] Liao W, Lin JX, Leonard WJ Interleukin-2 at the crossroads of effector responses, tolerance, and immunotherapy. Immunity. 2013;38:13-25. doi:10.1016/j.immuni.2013.01.004. PMID:23352221 23352221 PMC3610532

[55] Bao K, Reinhardt RL The differential expression of IL-4 and IL-13 and its impact on type-2 immunity. Cytokine. 2015;75:25-37. doi:10.1016/j.cyto.2015.05.008. PMID:26073683 26073683 PMC5118948

[56] Krissinel E, Henrick K Secondary-structure matching (SSM), a new tool for fast protein structure alignment in three dimensions. Acta Crystallogr D Biol Crystallogr. 2004;60:2256-68. doi:10.1107/S0907444904026460. PMID:15572779 15572779

[57] Pawig L, Klasen C, Weber C, Bernhagen J, Noels H Diversity and inter-connections in the CXCR4 chemokine Receptor/Ligand family: Molecular perspectives. Front Immunol. 2015;6:429. doi:10.3389/fimmu.2015.00429. PMID:26347749 26347749 PMC4543903

[58] Duensing TD, Wing JS, van Putten JP Sulfated polysaccharide-directed recruitment of mammalian host proteins: A novel strategy in microbial pathogenesis. Infect Immun. 1999;67:4463-8. PMID:10456887 10456887 10.1128/iai.67.9.4463-4468.1999 PMC96765

[59] Emsley P, Lohkamp B, Scott WG, Cowtan K Features and development of coot. Acta Crystallogr D Biol Crystallogr. 2010;66:486-501. doi:10.1107/S0907444910007493. PMID:20383002 20383002 PMC2852313

